# Alterations of Monetary Reward and Punishment Processing in Chronic Cannabis Users: An fMRI Study

**DOI:** 10.1371/journal.pone.0119150

**Published:** 2015-03-23

**Authors:** Björn Enzi, Silke Lissek, Marc-Andreas Edel, Martin Tegenthoff, Volkmar Nicolas, Norbert Scherbaum, Georg Juckel, Patrik Roser

**Affiliations:** 1 Department of Psychiatry, Psychotherapy and Preventive Medicine, LWL-University Hospital, Ruhr-University Bochum, Bochum, Germany; 2 Department of Neurology, BG University Hospital Bergmannsheil, Ruhr-University Bochum, Bochum, Germany; 3 Department of Radiology, BG University Hospital Bergmannsheil, Ruhr-University Bochum, Bochum, Germany; 4 Department of Addictive Behavior and Addiction Medicine, LVR Hospital, University of Duisburg-Essen, Essen, Germany; University of Groningen, NETHERLANDS

## Abstract

Alterations in reward and punishment processing have been reported in adults suffering from long-term cannabis use. However, previous findings regarding the chronic effects of cannabis on reward and punishment processing have been inconsistent. In the present study, we used functional magnetic resonance imaging (fMRI) to reveal the neural correlates of reward and punishment processing in long-term cannabis users (*n* = 15) and in healthy control subjects (*n* = 15) with no history of drug abuse. For this purpose, we used the well-established Monetary Incentive Delay (MID) task, a reliable experimental paradigm that allows the differentiation between anticipatory and consummatory aspects of reward and punishment processing. Regarding the gain anticipation period, no significant group differences were observed. In the left caudate and the left inferior frontal gyrus, cannabis users were – in contrast to healthy controls – not able to differentiate between the conditions feedback of reward and control. In addition, cannabis users showed stronger activations in the left caudate and the bilateral inferior frontal gyrus following feedback of no punishment as compared to healthy controls. We interpreted these deficits in dorsal striatal functioning as altered stimulus-reward or action-contingent learning in cannabis users. In addition, the enhanced lateral prefrontal activation in cannabis users that is related to non-punishing feedback may reflect a deficit in emotion regulation or cognitive reappraisal in these subjects.

## Introduction

Cannabis is one of the most widely used illicit drugs worldwide [1]. A variety of animal studies demonstrated that cannabis may affect the reward system thereby accounting for its addictive potential [2]. The neural network associated with reward processing includes the ventral tegmental area (VTA), limbic structures, particularly the ventral striatum, and frontal regions [3]. The acute rewarding effects of drugs of abuse are mediated by an elevation of mesolimbic dopaminergic neurotransmission in the nucleus accumbens, whereas chronic and compulsive drug use is associated with a hypodopaminergic reward system [4]. This so-called reward deficiency hypothesis might finally explain why subjects with addictive disorders tend to gradually increase their drug consumption in order to reach the same rewarding effect of well-being [5].

The psychoactive properties of cannabis are primarily produced by the plant cannabinoid Δ^9^-tetrahydrocannabinol (THC) and its interaction with the cannabinoid receptor type 1 (CB1) in the central nervous system [6]. Of note, CB1 receptors have been detected in brain regions associated with reward processing, including the VTA, striatum and prefrontal cortex [7]. Cannabinoid receptors and their endogenous ligands form the so-called endocannabinoid system that plays an important modulatory role for GABAergic, glutamatergic and dopaminergic neurotransmission [8].

With regard to reward processing, activation of the endocannabinoid system by acute exposure to THC, like other drugs of abuse, has been found to increase dopaminergic transmission in the nucleus accumbens in rats [9]. Similar results have been obtained for the endogenous cannabinoid receptor ligand anandamide [10]. In analogy to the aforementioned animal studies, acute administration of THC also induced dopamine release in the ventral striatum in humans as assessed by [^11^C]raclopride positron emission tomography (PET) [11], although this result could not be confirmed by another PET and a [^123^I]IBZM single photon emission tomography study [12,13]. However, a recent functional magnetic resonance imaging (fMRI) study demonstrated no changes in brain activity to monetary reward anticipation in healthy human subjects after acute administration of THC compared to placebo, but a widespread attenuation of brain activity to reward feedback [14]. On the other hand, inhibition of the endocannabinoid system by the CB1 receptor antagonist rimonabant has been shown to reduce the neural response to reward in animals [15] as well as in healthy humans [16]. These findings indicate that the endocannabinoid system appears to be involved in brain reward processes and drug addiction through the activation of the mesolimbic dopaminergic system.

Interestingly, chronic cannabis use has been associated with a decrease of dopaminergic activity in brain areas relevant for reward processing [2], presumably due to a down-regulation and desensitization of CB1 receptors under long-term exposure to cannabis [17]. This hypodopaminergia-induced reward deficiency might finally be responsible for the reduced activation of the striatum during monetary reward anticipation in regular cannabis users [18]. However, another recent fMRI study demonstrated an increase of ventral striatal activity during non-drug reward anticipation in chronic cannabis users with life-time cannabis use and quantity of life-time use being negatively correlated with ventral striatal BOLD response [19]. Despite these contradictory findings, there were no significant differences between cannabis users and non-using controls in reward feedback activity in both studies.

The monetary incentive delay (MID) task is a well-established paradigm to assess reward processing including the brain activity to reward anticipation as well as reward feedback during fMRI [20]. In the MID task, reward anticipation is typically associated with an increased activity in the ventral striatum, while reward feedback primarily activates frontal regions [21,22]. Dysfunction of reward processing has been shown by using this task for patients with alcohol [23] as well as nicotine dependence [24]. Apart from addiction, other major psychiatric disorders including major depression and schizophrenia have been linked to a dysfunctional reward system [25,26]. It is suggested that these findings are associated with anhedonia as a characteristic symptom of addictive, affective and psychotic diseases [27].

Therefore, the aim of the present study was to investigate the effects of chronic cannabis abuse on the processing of monetary reward and punishment and its significance for addiction development. The blood concentrations of THC and its metabolites have been investigated for correlations with the BOLD response to monetary reward.

## Methods

### Ethics statement

The presented study was approved by the ethics committee of the Ruhr-University Bochum, Germany. After a detailed explanation of the study, all subjects gave their written informed consent.

### Subjects

We investigated 15 cannabis users (all male, average age 26.33 ± 2.94 years, range 23 to 31 years) and 15 healthy controls (all male, average age 27.13 ± 3.85 years, range 19 to 33 years) with no history of psychiatric, neurological or medical illnesses. The control group was matched with regard to gender, age, intelligence, education, and smoking habits. To exclude a clinically relevant depression in our study group, all subjects completed the Beck-Depression-Inventory (BDI). The MWT-B was used as a measure of verbal intelligence. All cannabis users were interviewed regarding their current substance use pattern. In addition, 14 of 15 cannabis users provided blood samples for quantification of 11-nor-9-carboxy-Δ^9^-tetrahydrocannabinol (THC-COOH), the main non-psychotropic metabolite of THC, according to standard procedures [28]. Given its relatively long half-life of up to weeks in heavy cannabis users, THC-COOH was determined in order to estimate the extent of recent use. Further drug use was ruled out by urine drug screen. All subjects were instructed to refrain from alcohol and drug use (except nicotine and caffeine) for 24 hours. A detailed description of the study population is given in Table 1.

**Table 1:** Characteristics of healthy controls and cannabis misuse subjects.

	Healthy (*n* = 15)	Cannabis (*n* = 15)	statistics
Age (years; ± SD)	27.13 (± 3.85)	26.33 (± 2.94)	*p* = 0.528^1^
Sex (male/female)	15/0	15/0	—
Education years (mean; ± SD)	18.39 (± 3.09)	17.77 (± 2.76)	*p* = 0.569^1^
Intelligence score [MWT-B] (mean; ± SD)	115.27 (± 14.06)	108.93 (± 8.95)	*p* = 0.152^1^
BDI score (mean; ± SD)	3.54 (± 2.4)	5.93 (± 3.9)	*p* = 0.066^1^
Cigarettes per day (mean; ± SD)	8 (± 9.2)	14.8 (± 13.26)	*p* = 0.098^2^
Alcohol [standard drinks/week] (mean; ± SD)	3.8 (± 1.7)	5.87 (± 3.72)	p = 0.056^2^
THC-COOH [ng/ml] (mean; ± SD)	NA	86.84 (± 112.68)^3^	—
Cannabis use age onset [years](mean; ± SD)	NA	15.87 (± 2.7)	—
Cannabis use [years] (mean; ± SD)	NA	8.47 (± 2.97)	—
Actual cannabis use [joints per week] (mean; ± SD)	NA	13.27 (± 7.28)	—
Abstinence [days] (mean; ± SD)	NA	1.1 (± 1.06)	—
Mean RT for ‘no outcome’ (ms; ± SD)	243.04 (± 53.47)	231.56 (± 32.53)	*p* = 0.511^1^
Mean RT for ‘reward’ (ms; ± SD)	208.67 (± 24.47)	210.26 (± 24.0)	*p* = 0.859^1^
Mean RT for ‘punishment’ (ms; ± SD)	213.09 (± 26.59)	207.78 (± 20.86)	*p* = 0.528^1^

^1^ *t*-test for independent samples, two-sided;

^2^ Mann-Whitney-*U* test;

^3^ *n* = 13, one subject’s data is missing due to insufficient blood samples, one subject showed a concentration < 1,5 ng/ml.

*Abbreviations*: BDI: Beck Depression Inventory; NA: not applicable; MWT-B: *Mehrfachwortschatzintelligenztest* [score for verbal intelligence]; RT: reaction time; SD: standard deviation; THC-COOH: 11-nor-9-carboxy-delta-9-tetrahydrocannabinol.

### Experimental paradigm

We used the MID task [20,29], a well-established experimental paradigm for investigating the anticipatory and consummatory aspects of reward and punishment processing. Before scanning, all subjects completed a short practice version of the task to familiarize them fully with the experiment.

**Fig 1:**
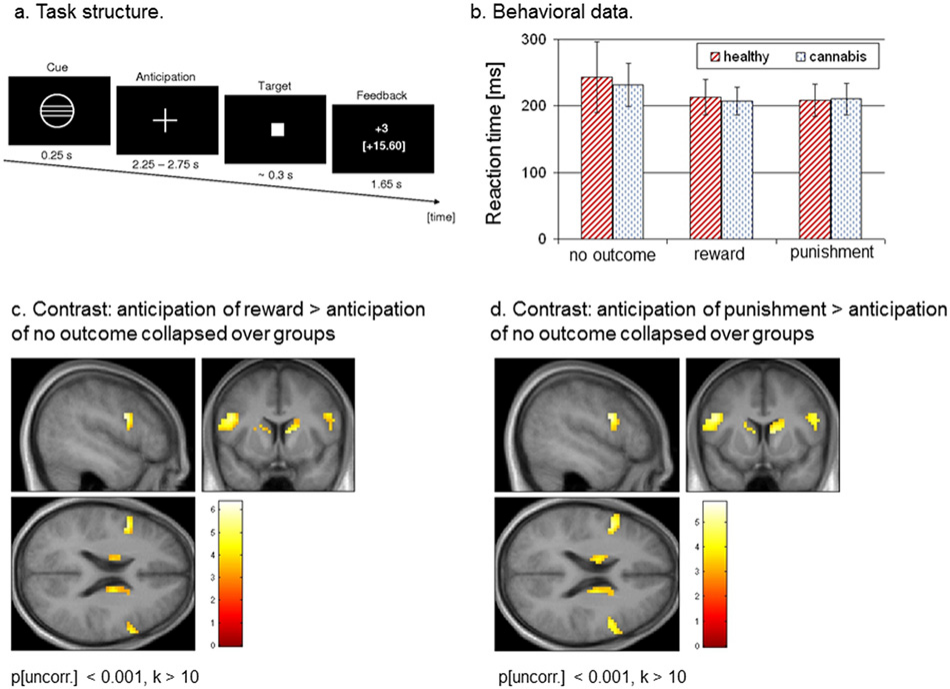
Task design, behavioural data, and fMRI results. a. Design and schematic structure of the applied fMRI paradigm. b. Reaction times for the conditions reward, punishment, and no outcome in healthy subjects and cannabis users. c. Contrast ‘anticipation of reward > anticipation of no outcome’ collapsed over both groups. The initial threshold was set to p[uncorr.] < 0.001 for k > 10. d. Contrast ‘anticipation of punsihment > anticipation of no outcome’ collapsed over both groups. The initial threshold was set to p[uncorr.] < 0.001 for k > 10.

In each trial, subjects were presented with a cue indicating what the possible outcomes of the task would be, i.e., reward, punishment, or no outcome, followed by a 3740–4240 ms anticipation period. In total, we used six different cues signalling the amount of potential monetary gain or loss (10 ct, 60 ct or 3 €), the no outcome trials served as control condition. The MID task requires the subject to press a button with the index finger of their right hand within a certain time of a target image (a square in the centre of the screen) being displayed. The length of this time period was individually adapted according to the subjects’ actual task performance, ensuring that in approximately ^2^/_3_ of all trials the required response was successful. In our case, the target duration varied from 160 ms to 360 ms followed by a 1500–2200 ms delay period. Subjects were instructed to respond to the target as quickly as possible. In total, 54 reward and punishment trials and 36 no outcome trials were displayed in a pseudorandomized order. Each trial was followed by a 1650 ms feedback period during which the subject was informed of the outcome. According to the above mentioned structure of the MID task, it is possible to distinguish four possible anticipation-feedback combinations: (1) reward cue and rewarding feedback after successful performance (hereinafter referred to as ‘fdb rew’), (2) reward cue and non-rewarding feedback after unsuccessful performance, (3) punishment cue and non-punishing feedback after successful performance (hereinafter referred to as ‘fdb pun’), and (4) punishment cue and punishing feedback after unsuccessful performance. All trials were separated by a 4 s inter-trial interval (for a more detailed description of the MID task see Enzi et al., 2012 and Fig. 1). The experiment was presented via MRI-compatible LCD goggles (Resonance Technology Inc., Los Angeles, CA, USA) using the Presentation 11.3 software package (Neurobehavioral Systems Inc., Albany, CA, USA). All subjects were paid according to their performance in the MID task.

### fMRI data acquisition and analysis

Functional data was collected using a 1.5-Tesla whole body MRI system (Siemens Magnetom Symphony, Erlangen, Germany) equipped with a high power gradient system (30 mT/m/s; SR 125 T/m/s) and a standard imaging head coil. 22 T2*-weighted echo-planar (EPI) images per volume with blood-oxygen level dependent (BOLD) contrast were obtained using a gradient-echo sequence (GE-EPI; TR = 1900 ms, TE = 45 ms, flip angle α = 90°, matrix 64 x 64, spatial resolution 3.8 x 3.8 x 3.8 mm^3^). The slices were acquired parallel to the bi-commissural plane and covered striatal and prefrontal regions of interest [21]. Subjects had to complete two scanning runs with 450 volumes per run. The first three volumes were discarded due to saturation effects. Prior to the functional scanning session, a T1-weighted anatomical 3D magnetization prepared rapid gradient echo scan (MP-RAGE; TR = 9.7 ms, TE = 4 ms, flip angle α = 12°, matrix 256 x 256, spatial resolution 1 x 1 x1 mm^3^) was acquired for each subject.

The functional data was preprocessed and statistically analysed using the SPM8 software package (Wellcome Department of Cognitive Neuroscience, University College London, UK; http://www.fil.ion.ucl.ac.uk) and MATLAB 7.11 (The Mathworks Inc, Natick, MA, USA). After temporal correction and correction for between-scan motion artefacts by realignment to the first volume, the anatomical scan was coregistered to a mean functional image. The normalization was generated by warping the subject’s anatomical T1-weighted scan on the T1-template provided by SPM5 (MNI stereotactic space) and applying these parameters to all functional images. The images were resampled to a final voxel size of 3 x 3 x 3 mm^3^ and smoothed with an isotropic 8 mm full-width half-maximum Gaussian kernel. The time-series fMRI data was filtered using a high-pass filter with a 128 s cut-off. Functional runs with translational movement greater 2 mm and/or rotational movement greater 1° were excluded from statistical analysis. For this reason, we discarded the 2^nd^ functional scanning run of two healthy subjects and two cannabis users.

For the MID task, all relevant conditions, i.e., anticipation of reward, anticipation of punishment, anticipation of no outcome and their feedback phase according to successful task performance were modelled, resulting in six conditions. Additionally, the six realignment parameters were entered as regressors of no interest. A statistical model for each subject was computed by convolving a canonical hemodynamic response function with the above-mentioned design [30]. All further statistical analyses followed the general linear model approach [31]. Regionally specific condition effects were tested by employing linear contrasts for each subject and each condition of interest. Two separate second-level models for the anticipation period and the feedback period were calculated using the full factorial-option implemented in SPM8 with the factors ‘group’ (healthy, cannabis users) and ‘task’ (separately for the anticipation and feedback period for the conditions reward, punishment, and no outcome). Only activations surviving family-wise error correction on voxel-level (p < 0.05) were reported. Small volume correction (S.V.C., radius 5 mm) was used where appropriate. The anatomical localization of significant activations was assessed with reference to a standard stereotactic atlas [32] by superimposition of the SPM maps on an averaged brain of all subjects.

Using sphere-shaped regions of interest (ROI; radius 5 mm) centred upon the peak voxel within each area of interest, signal changes (expressed in percent) for the conditions of interest, i.e., successful performance in reward, punishment and no outcome trials, were extracted using the “rfxplot”-toolbox (http://rfxplot.sourceforge.net/) for SPM [33].

All further statistical analyses (*t*-tests for dependent and independent samples, nonparametric Mann-Whitney-*U* test, Kolmogorov-Smirnov test, Pearson correlation) were calculated using the software package SPSS 18 (SPSS Inc., Chicago, USA).

## Results

### Demographic, clinical and behavioural data

Healthy subjects and cannabis users did not differ significantly with regard to age, sex, intelligence, education years and smoking habits. Regarding the MID task, we were not able to detect a significant group difference in reaction times for the conditions reward, punishment and no outcome. Further statistical details concerning the demographic, clinical and behavioral data are given in Table 1 and Fig. 1b.

### Functional imaging data

#### Activations in response to anticipation of reward

We first investigated the activation pattern concerning the contrasts ‘anticipation of reward > anticipation of no outcome’ collapsed over groups and ‘anticipation of punishment > anticipation of no outcome’ collapsed over groups. As expected, the above-mentioned contrasts revealed a set of brain regions typically involved in reward and punishment processing, e.g., the bilateral striatum, the bilateral inferior frontal gyrus, and the bilateral anterior insula. In addition, the interaction contrast between the factors ‘group’ [healthy, cannabis users] and ‘task anticipation’ [ant rew, ant pun, ant noc] showed no significant activations related to the anticipation period. (Fig. 1 and Table 2).

**Table 2:** List of activations related to the anticipation period.

	Region	Coordinates [MNI]	Cluster size [voxel]	p[FWE] on voxel-level	t-value
f-contrast interaction group [healthy, cannabis] x condition [ant rew, ant pun, ant noc]					
	No significant activations at p[uncorr.] < 0.001 for k > 10.				
t-contrast [anticipation of reward > anticipation of punishment] collapsed over groups					
	No significant activations at p[uncorr.] < 0.001 for k > 10.				
t-contrast [anticipation of punishment > anticipation of reward] collapsed over groups					
	No significant activations at p[uncorr.] < 0.001 for k > 10.				
t-contrast [anticipation of reward > anticipation of no outcome] collapsed over groups					
L	Striatum/caudate nucleus	-12, -1, 13	54	0.003	4.74
R	Striatum/caudate nucleus^1^	12, 8, 10	99	0.001	5.23
R	Anterior insula	33, 17, 10		0.035	3.98
L	Anterior Insula	-33, 20, 10	44	< 0.001	6.36
L	Inferior frontal gyrus	-48, 5, 25	76	< 0.001	5.88
R	Inferior frontal gyrus	51, 8, 28	45	0.021	4.15
t-contrast [anticipation of punishment > anticipation of no outcome] collapsed over groups					
L	Striatum/caudate nucleus	-9, 8, 10	65	0.046	3.88
R	Striatum/caudate nucleus^1^	12, 11, 10	136	0.001	5.04
R	Anterior insula	33, 20, 10		0.009	5.0
L	Anterior insula	-36, 23, 10	46	< 0.001	5.8
L	Inferior frontal gyrus	-48, 5, 25	100	< 0.001	5.35
R	Inferior frontal gyrus	54, 8, 28	60	0.012	4.33
t-contrast: [anticipation of reward > no outcome] in [healthy > cannabis users] et vice versa					
	No significant activations at p[uncorr.] < 0.001 for k > 10.				
t-contrast [anticipation of punsihment > no outcome] in [healthy > cannabis users] et vice versa					
	No significant activations at p[uncorr.] < 0.001 for k > 10.				

The initial threshold was set to *p*[uncorr.] < 0.001 for an extent *k* > 10 voxel.

^1^ extending to the right anterior insula.

#### Activations in response to feedback of reward and punishment

**Fig 2:**
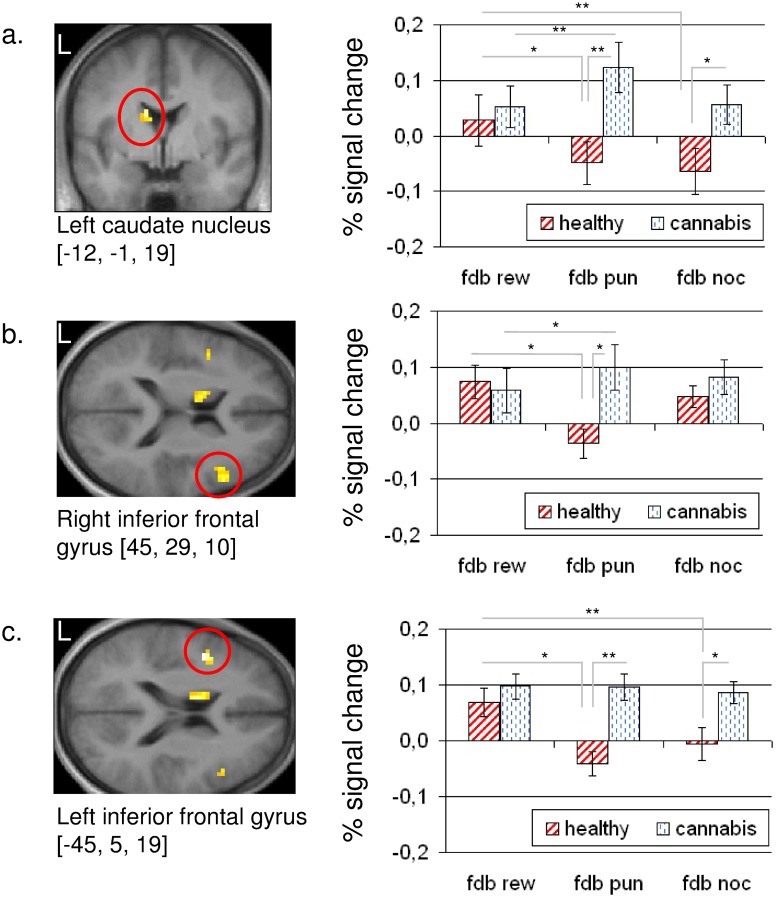
fMRI data regarding the feedback period. Statistical parametric maps representing the interaction contrast between ‘group’ (healthy, cannabis users) and ‘task feedback’ (fdb rew, fdb pun, fdb noc). Percent signal change derived from the (a) left caudate nucleus, (b) right inferior frontal gyrus, and (c) left inferior frontal gyrus. All regions of interest are circled in red. All statistical parametric maps are thresholded at p[uncorr] < 0.001 for k > 10. ** p < 0.01; * p < 0.05. Error bar represents SEM.

For assessment of group differences regarding the feedback conditions after successful task performance, the interaction f-contrast between ‘group’ [healthy, cannabis users] and ‘task feedback’ [fdb rew, fdb pun, fdb noc] was calculated. This contrast revealed significant differences in activation between control subjects and cannabis users located in the left caudate nucleus and the bilateral inferior frontal gyrus (Fig. 2).

In the left caudate nucleus (MNI: -12, -1, 19), healthy subjects showed a significant differentiation between the conditions ‘fdb rew’ and ‘fdb pun’ (t_14_ = 4.357; p = 0.001) and between ‘fdb rew’ and ‘fdb noc’ (t_14_ = 2.294; p = 0.038), whereas cannabis users showed only a significant differentiation between the conditions ‘fdb rew’ and ‘fdb pun’ (t_14_ = 3.042; p = 0.009). Compared to healthy controls, cannabis users showed an increased activation of the left caudate regarding the conditions ‘fdb pun’ (t_28_ = 2.914; p = 0.007) and ‘fdb noc’ (t_28_ = 2.213; p = 0.035).

A similar activation pattern was observable in the bilateral inferior frontal gyrus, opercular part. In the right inferior frontal gyrus (MNI: 45, 29, 10), both groups were able to differentiate significantly between the conditions ‘fdb rew’ and ‘fdb pun’ (healthy: t_14_ = 2.705; p = 0.017; cannabis users: t_14_ = 2.232; p = 0.042), but compared to healthy controls cannabis users showed an increased activation concerning the condition ‘fdb pun’ (t_28_ = 2.747; p = 0.01). In the left inferior frontal gyrus (MNI: -45, 5, 19), only control subjects were able to differentiate between ‘fdb rew’ and ‘fdb pun’ (t_14_ = 4.828; p < 0.001) and between ‘fdb rew’ and ‘fdb noc’ (t_14_ = 2.554; p = 0.023), respectively. In contrast to healthy subjects showing a deactivation regarding the conditions ‘fdb pun’ and ‘fdb noc’, an increased response was observed in cannabis users for the very same conditions (‘fdb pun’: t_28_ = 4.317; p < 0.001; ‘fdb noc’: t_28_ = 2.598; p = 0.015) (Table 3, Fig. 2).

**Table 3:** List of activations related to the feedback period.

	Region	Coordinates [MNI]	Cluster size [voxel]	p[FWE]	Statistical value
f-contrast: interaction group [healthy, cannabis] x condition [fdb rew, fdb pun, fdb noc]					
L	Caudate nucleus	-12, -1, 19	23	0.005^3^	8.71^1^
L	Inferior frontal gyrus	-45, 5, 19	22	0.001^3^	10.44^1^
R	Inferior frontal gyrus	45, 29, 10	85	0.004^3^	9.04^1^
t-contrast: [feedback of reward > feedback of punishment] collapsed over groups					
L	Inferior frontal gyrus	-48, 8, 25	47	< 0.001	5.87^2^
R	Inferior frontal gyrus	51, 8, 28	70	0.015	4.29^2^
t-contrast: [feedback of punishment > feedback of reward] collapsed over groups					
	No significant activations at p[uncorr.] < 0.001 for k > 10.				
t-contrast: [feedback of reward > feedback of no outcome] collapsed over groups					
	No significant activations at p[uncorr.] < 0.001 for k > 10.				
t-contrast: [feedback of reward > feedback of no outcome] collapsed over groups					
	No significant activations at p[uncorr.] < 0.001 for k > 10.				

The initial threshold was set to *p*[uncorr.] < 0.001 for an extent *k* > 10 voxel.

^1^: f-value;

^2^: t-value;

^3^: with small volume correction (S.V.C., radius 5 mm)

#### Correlation results

**Fig 3:**
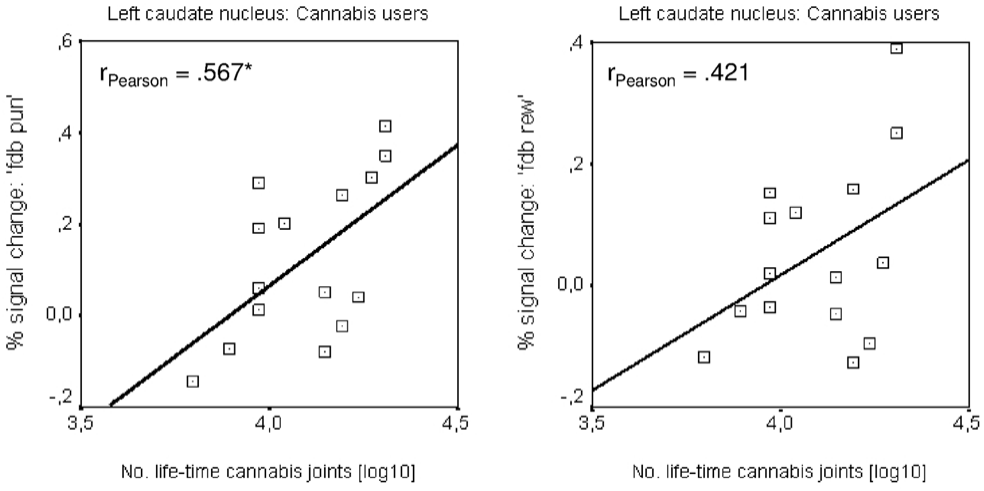
Correlation results. Correlation between life-time cannabis use indicated by the life-time number of cannabis joints (log-transformed) and BOLD activity derived from the left dorsal caudate during the feedback period for the conditions reward and punishment. * p < 0.05.

Pearson correlations were calculated to explore the relationship between the number of life-time cannabis joints (log-transformed) and the conditions ‘fdb pun’ and ‘fdb rew’, respectively. The correlation analysis was restricted to the left caudate nucleus. In this context, we observed a significant positive correlation (p < 0.05) between the number of life-time cannabis joints and ‘fdb pun’, whereas between ‘fdb rew’ and life time cannabis use a non-significant result was obtained (Fig. 3). All other correlations (between the fMRI signal and THC-COOH, and between the fMRI signal and cannabis use age onset, actual cannabis use and abstinence) showed non-significant results.

## Discussion

In the present study we investigated the neural correlates of anticipatory and consummatory aspects of reward and punishment processing in cannabis users and healthy control subjects. Both groups showed reliable activations of the reward circuit associated with the anticipation of gain and punishment. However, no significant group differences regarding gain or punishment anticipation were observable. During the gain anticipation period, both groups, i.e. cannabis users and healthy controls, showed a brain activation pattern commonly associated with reward and punishment processing, including the ventral striatum, the anterior insula, and the inferior frontal gyrus [3,20].

In the left caudate and the left inferior frontal gyrus, cannabis users showed deficits in the differentiation between the conditions feedback of reward and control, a neural differentiation between feedback of reward and feedback of punishment was only observed in the left caudate nucleus and the right inferior frontal gyrus.

We were not able to replicate previous findings of an altered ventral striatal BOLD activity during the anticipation of reward in cannabis users [18,19]. A possible explanation for this results may arise from the fact that the cannabis users investigated by Nestor and colleagues [19] showed a longer period of abstinence before fMRI (approximately 108 hours vs. 24 hours for the group investigated in the present study). This explanation gains further support from a recent fMRI study using the MID task after acute administration of Δ^9^-THC in healthy volunteers [14]. The authors report an attenuated neural response in various brain regions related to gain feedback whereas the “anticipatory brain activity was not affected” [14].

The striatum, mainly the ventral striatum (VS), plays a key role in reward processing in animals and humans and is part of the so-called reward system, a complex network including the ventral tegmental area, the ventral putamen, the anterior cingulate cortex, and the orbitofrontal cortex. Important regulating structures of the reward system are the lateral prefrontal cortex, the amygdala, hippocampus, thalamus and specific brainstem nuclei [3]. Recent research suggests that the dorsal striatum plays an important role in reward processing and decision-making by linking rewarding outcomes to subsequent behaviour [34,35], and thus associating “reward to action” [36]. In this context, obtaining a reward requires learning of stimulus-response or stimulus-reward associations [37], and this so-called “action-contingent learning” about the rewarding properties of an action has been found to depend on dorsal striatal functioning [34]. Therefore, the lack of neural differentiation between ‘fdb rew’ and ‘fdb noc’ observed in the left caudate nucleus probably reflects an impaired action-contingent or stimulus-response-reward learning in cannabis users. Since the behavioural measures of the above described learning process, i.e. the reaction times, are not affected in cannabis users, the observed activation of the caudate nucleus may reflect a compensatory mechanism ensuring adequate task performance. In addition, subjects with the highest degree of cannabis use showed a more increased activation of the caudate nucleus in response to the condition ‘fdb pun’, suggesting that altered action-contingent learning is associated with life-time cannabis use. In all three regions investigated in this study, i.e. the left caudate nucleus and the bilateral inferior frontal gyrus, cannabis users showed increased activations regarding the condition ‘fdb pun’ compared to healthy controls. Since our condition ‘fdb pun’ reflects a non-punishing outcome after successful task performance—commonly called negative reinforcement, i.e. negative outcome avoidance [35]—this result reflects an enhanced neural response related to loss-avoidance in cannabis users. The inferior frontal gyrus is an important part of the lateral prefrontal cortex (PFC), a brain region commonly associated with cognitive and emotional processing [38]. In more detail, the lateral PFC is involved in emotion regulation, i.e., “changing the onset, duration, intensity or content of an emotional response” [39], or—more specifically—cognitive reappraisal, i.e., the cognitve reinterpretation of (emotional) information for changing an emotional response [40]. A common experimental paradigm for investigating cognitive reappraisal consists of several negatively valenced emotional pictures and requires the participant to cognitively re-interpret “meaning, cause, consequence or personal significance” of the shown picture [39]. Cognitive reappraisal is associated with activation of the bilateral dorsolateral PFC, the bilateral ventrolateral PFC including the inferior frontal gyrus, the dorsal anterior cingulate cortex and the medial PFC [39], a set of regions associated with cognitive suppressive ‘top-down’ control of the subcortical limbic and paralimbic system. The activation of the bilateral inferior frontal gyrus associated with non-punishing outcomes after sufficient task-response could therefore reflect a deficit in emotion regulation and/or cognitive reappraisal in cannabis users. Following Ochsner and Gross [40], the ventral PFC and the orbitofrontal cortex play a key role in the evaluation of stimuli regarding their “context-appropriate emotional value” and in the selection of actions based on these evaluations. The observed increased activation of the bilateral inferior frontal gyrus may reflect a higher emotional involvement related to loss-avoidance in cannabis users, whereas healthy subjects show an increased neural response associated with reward delivery. In the left caudate nucleus and the left inferior frontal cortex, the above-mentioned feedback-related alterations in cannabis users found for the condition ‘fdb pun’ were accompanied by an increased neural response associated with the control condition. Since only successful trials, i.e. trials where the subjects responded within a defined time period, were entered as regressors in the design, this activation pattern in cannabis users could be related to altered performance monitoring in general, and thus independent of the reward and punishment.

Finally, we want to discuss two important limitations of the present study. (1) Since the sample size per group (n = 15) is relatively small, we suggest to consider our results as preliminary. Nevertheless, it should be noted that various fMRI studies in cannabis users reported similar sample sizes. (2) Due to technical limitations of the used MRI system we were not able to cover all brain regions associated with emotion and/or reward processing, like e.g. the amygdala, the gyrus parahippocampalis, and the entorhinal gyrus.
